# Interim 4′-[methyl-^11^C]-thiothymidine PET for predicting the chemoradiotherapeutic response in head and neck squamous cell carcinoma: comparison with [^18^F]FDG PET

**DOI:** 10.1186/s13550-021-00749-y

**Published:** 2021-02-10

**Authors:** Katsuya Mitamura, Takashi Norikane, Yuka Yamamoto, Kengo Fujimoto, Yasukage Takami, Hiroshi Hoshikawa, Jun Toyohara, Yoshihiro Nishiyama

**Affiliations:** 1 Department of Radiology, Faculty of Medicine, Kagawa University, 1750-1 Ikenobe, Miki-cho, Kita-gun, Kagawa, 761-0793 Japan; 2 Department of Otolaryngology, Faculty of Medicine, Kagawa University, Kagawa, Japan; 3 Research Team for Neuroimaging, Tokyo Metropolitan Institute of Gerontology, Tokyo, Japan

**Keywords:** [^11^C]4DST, [^18^F]FDG, PET, Squamous cell carcinoma

## Abstract

**Purpose:**

We investigated the potential of interim 4′-[methyl-^11^C]thiothymidine ([^11^C]4DST) PET for predicting the chemoradiotherapeutic response for head and neck squamous cell carcinoma (HNSCC), in comparison with 2-deoxy-2-[^18^F]fluoro-D-glucose ([^18^F]FDG) PET.

**Methods:**

A total of 32 patients with HNSCC who underwent both [^11^C]4DST and [^18^F]FDG PET/CT before therapy (baseline) and at approximately 40 Gy point during chemoradiotherapy (interim) were available for a retrospective analysis of prospectively collected data. The baseline was treatment-naïve PET/CT scan as part of staging. The maximum standardized uptake value (SUVmax), metabolic tumor volume (MTV) from [^18^F]FDG PET or proliferative tumor volume (PTV) from [^11^C]4DST PET, and total lesion glycolysis (TLG) from [^18^F]FDG PET or total lesion proliferation (TLP) from [^11^C]4DST PET were measured. MTV or PTV was defined as the volume with an SUVmax greater than 2.5. The differences in SUVmax (ΔSUVmax), MTV (ΔMTV) or PTV (ΔPTV) and TLG (ΔTLG) or TLP (ΔTLP) from baseline to interim PET scans were calculated. Patients without or with evidence of residual or recurrent disease at 3 months after completion of chemoradiotherapy were classified as showing a complete response (CR) and non-CR, respectively.

**Results:**

All patients showed increased uptake in primary tumor on baseline [^11^C]4DST and [^18^F]FDG PET studies. All patients showed increased uptake on interim [^18^F]FDG PET, whereas 18 patients showed no increased uptake on interim [^11^C]4DST PET. After chemoradiotherapy, 25 patients were found to be in CR group and 7 to be in non-CR group. [^11^C]4DST ΔSUVmax, ΔPTV, and ΔTLP for CR group showed significantly greater reductions than the corresponding values for non-CR group (*P* = 0.044, < 0.001, < 0.001, respectively). However, there were no significant differences in [^18^F]FDG ΔSUVmax, ΔMTV, or ΔTLG between CR group and non-CR group. [^11^C]4DST ΔMTV of -90 was the best cutoff value for the early identification of patients with non-CR.

**Conclusion:**

These preliminary results suggest that interim [^11^C]4DST PET might be useful for predicting the chemoradiotherapeutic response in patients with HNSCC, in comparison with [^18^F]FDG PET.

## Introduction

Concurrent chemoradiotherapy plays a major role in the management of locoregionally advanced head and neck squamous cell carcinoma (HNSCC) [1]. Accurate early evaluation of the therapeutic response is important to avoid ineffective treatments and unnecessary side effects. Positron emission tomography (PET) with 2-deoxy-2-[^18^F]fluoro-D-glucose ([^18^F]FDG) is a valuable functional imaging modality for diagnosis and follow-up of HNSCC [2]. Although [^18^F]FDG PET is valuable for assessment of the therapeutic response, [^18^F]FDG also accumulates at inflammatory lesions, and so false-positive results may be obtained [3–5]. The optimal time to make an accurate evaluation has been thought to be 3–4 months after radiotherapy [4, 5]. However, it is favorable that the response to radiotherapy is evaluated as soon as possible because it is necessary to determine the need for salvage therapy. The appropriate timing of the early therapeutic response using [^18^F]FDG PET remains unclear.

[^18^F]FDG directly reflects the glucose metabolism. Toyohara et al. developed 4′-[methyl-^11^C] thiothymidine ([^11^C]4DST) for cell proliferation imaging that is resistant to degradation by thymidine phosphorylase and is incorporated into deoxyribonucleic acid (DNA) [6]. [^11^C]4DST has been found to be helpful for noninvasive evaluation of the proliferation of various types of tumor [7–9]. In patients with HNSCC, [^11^C]4DST PET was found to provide important prognostic information [9].

To the best of our knowledge, no report has been published focusing on [^11^C]4DST PET for early evaluation of treatment in patients with HNSCC. Therefore, we investigated the effectiveness of interim [^11^C]4DST PET for predicting the chemoradiotherapeutic response in patients with HNSCC, in comparison with [^18^F]FDG PET.

## Materials and methods

### Patients

We conducted a retrospective analysis of prospectively collected data. The prospective study consisted of 259 consecutive, untreated patients with primary head and neck tumors who underwent [^11^C]4DST PET/CT study between May 2011 and March 2020. All patients included gave written and informed consent and study protocol was approved by the institutional ethics committee of the Kagawa University (registration number: 23011). From these patients, 32 patients (29 males, 3 females; mean age, 63.3 years; age range, 51–74 years) were selected for this retrospective analysis. Eligible patients fulfilled all of the following criteria: (1) histologically confirmed squamous cell carcinoma of the head and neck region with treatment-naïve; (2) both [^11^C]4DST and [^18^F]FDG PET/CT performed before treatment (baseline); (3) received radical chemoradiotherapy after baseline PET/CT studies; and (4) both [^11^C]4DST and [^18^F]FDG PET/CT performed at approximately 40 Gy point during chemoradiotherapy (interim). This retrospective data collection was compliant with the institutional ethics committee of the Kagawa University, with a waiver of informed consent (registration number: 2020078). Some of the data from 18 of these patients were used in a previous study [9]. Their clinical data are summarized in Table 1.

**Table 1:** Patient clinical characteristics

Characteristic	Value
Age (years)	
Mean	63.3
Range	51–74
Sex (n)	
Male	29
Female	3
Lesion site (*n*)	
Nasopharynx	1
Oropharynx	8
Hypopharynx	16
Larynx	5
Oral cavity	2
T category (*n*)	
T2	23
T3	5
T4	4
N category (*n*)	
N0	7
N1	5
N2	19
N3	1
M category (*n*)	
M0	29
M1	3

### Treatment and response

Radiotherapy was administered to the primary head and neck regions once daily using 4–MV photons with a pair of bilaterally opposed fields in the upper neck and an anterior port at the lower neck. Patients were irradiated with a total dose of 62–70 Gy in 2 Gy fractions once daily. After administration of 40 Gy, the clinical target volume was reduced to encompass only the primary tumor and involved neck lymph nodes. All patients received 1–3 courses of systemic chemotherapy. Ten patients received chemotherapy with cisplatin (70 mg/m^2^) and 5-fluorouracil (1,000 mg/m^2^ continuous infusion for 5 days), and 20 patients received chemotherapy with nedaplatin (80 mg/m^2^) and S-1 (100 mg/day for 14 days). The remaining 2 patients with T2 laryngeal cancer received weekly docetaxel (10 mg/m^2^) chemotherapy 6 times during radiotherapy.

Response at 3 months after completion of chemoradiotherapy was clinically evaluated on the basis of naso-endoscopy (25 patients), CT (26 patients), magnetic resonance imaging (1 patient), [^11^C]4DST and [^18^F]FDG PET/CT (32 patients), and biopsy (2 patients). Patients without or with evidence of residual or recurrent disease at 3 months were classified as showing a complete response (CR) and non-CR, respectively, by the head and neck tumor board team meetings, prospectively.

### Radiotracer synthesis and PET/CT imaging

The radiotracers, [^11^C]4DST and [^18^F]FDG, were manufactured using an automated synthesis system with HM-18 cyclotron (QUPID; Sumitomo Heavy Industries Ltd, Tokyo, Japan). The [^11^C]4DST was synthesized using the method described by Toyohara et al. [6].

All acquisitions were performed using a Biograph mCT 64-slice PET/CT scanner (Siemens Medical Solutions USA Inc., Knoxville, TN, USA), which has an axial field of view of 21.6 cm. Interim PET/CT scans were obtained at approximately 40 Gy point during chemoradiotherapy (median 42 Gy; range 32–50 Gy). The median intervals between [^11^C]4DST and [^18^F]FDG PET/CT studies for baseline and interim scans were 5 days (range 0–70 days) and 1 day (range 0–44 days), respectively.

Patients fasted for at least 5 h prior to [^18^F]FDG administration, and a normal glucose level in the peripheral blood was confirmed prior to [^18^F]FDG injection. Emission data were acquired from the midcranium to the proximal thighs (2 min per bed position) at 15 min after intravenous injection of [^11^C]4DST (7.4 MBq/kg) and 90 min after intravenous injection of [^18^F]FDG (3.7 MBq/kg). Unenhanced, low-dose CT of the same area was performed for attenuation correction and image fusion. PET data were reconstructed with an ordered subset expectation maximization algorithm, incorporating correction with point-spread function (PSF) and time-of-flight model (2 iterations, 21 subsets) using a Gaussian filter. Quantification were based on PSF-reconstructed data.

### Image analyses

A board-certified nuclear medicine physician, who had 7 years of experience in reading [^11^C]4DST and [^18^F]FDG PET/CT, performed PET/CT image analyses retrospectively. PET/CT image were assessed on the presence of foci of increased activity within the primary tumor greater than surrounding background. The standardized uptake value (SUV) was calculated using the following formula: SUV = *c*_dc_/(*d*_i_/*w*), where *c*_dc_ is the decay-corrected tracer tissue concentration (Bq/g); *d*_i_, the injected dose (Bq); and *w*, the patient’s body weight (g). The maximum SUV (SUVmax) from both the [^18^F]FDG and [^11^C]4DST PET studies and metabolic tumor volume (MTV) from [^18^F]FDG PET or proliferative tumor volume (PTV) from [^11^C]4DST PET for primary tumor were measured. MTV or PTV was defined as the volume with an SUVmax greater than 2.5 and to exclude adjacent [^18^F]FDG-avid or [^11^C]4DST-avid structures [8, 9]. When no tumor-related radioactivity was discernible visually (interim PET studies), the mean SUV of the primary region on the basis of baseline PET studies was measured and MTV or PTV was assumed to be zero. Total lesion glycolysis (TLG) and total lesion proliferation (TLP) were calculated in the [^18^F]FDG and [^11^C]4DST PET studies, respectively, as follows: MTV or PTV × mean SUV. The differences in SUVmax (ΔSUVmax), MTV (ΔMTV) or PTV (ΔPTV) and TLG (ΔTLG) or TLP (ΔTLP) from baseline to interim PET scans were calculated using the following formula: ΔSUVmax = (interim SUVmax − baseline SUVmax) × 100/baseline SUVmax; ΔMTV = (interim MTV − baseline MTV) × 100/baseline MTV; ΔPTV = (interim PTV − baseline PTV) × 100/baseline PTV; ΔTLG = (interim TLG − baseline TLG) × 100/ baseline TLG; ΔTLP = (interim TLP − baseline TLP) × 100/ baseline TLP.

### Statistical analyses

The data were analyzed using SPSS statistical software (version 26; IBM). PET parameters of the baseline and interim scans were compared using the paired *t* test. PET parameters between CR and non-CR groups were compared using the Mann–Whitney *U* test. Receiver operating characteristics (ROC) analysis was performed to determine the effectiveness of PET parameters for differentiating the early chemoradiotherapeutic response. Two-tailed values of *P* < 0.05 were considered statistically significant.

## Results

### Baseline and interim PET/CT

**Fig. 1:**
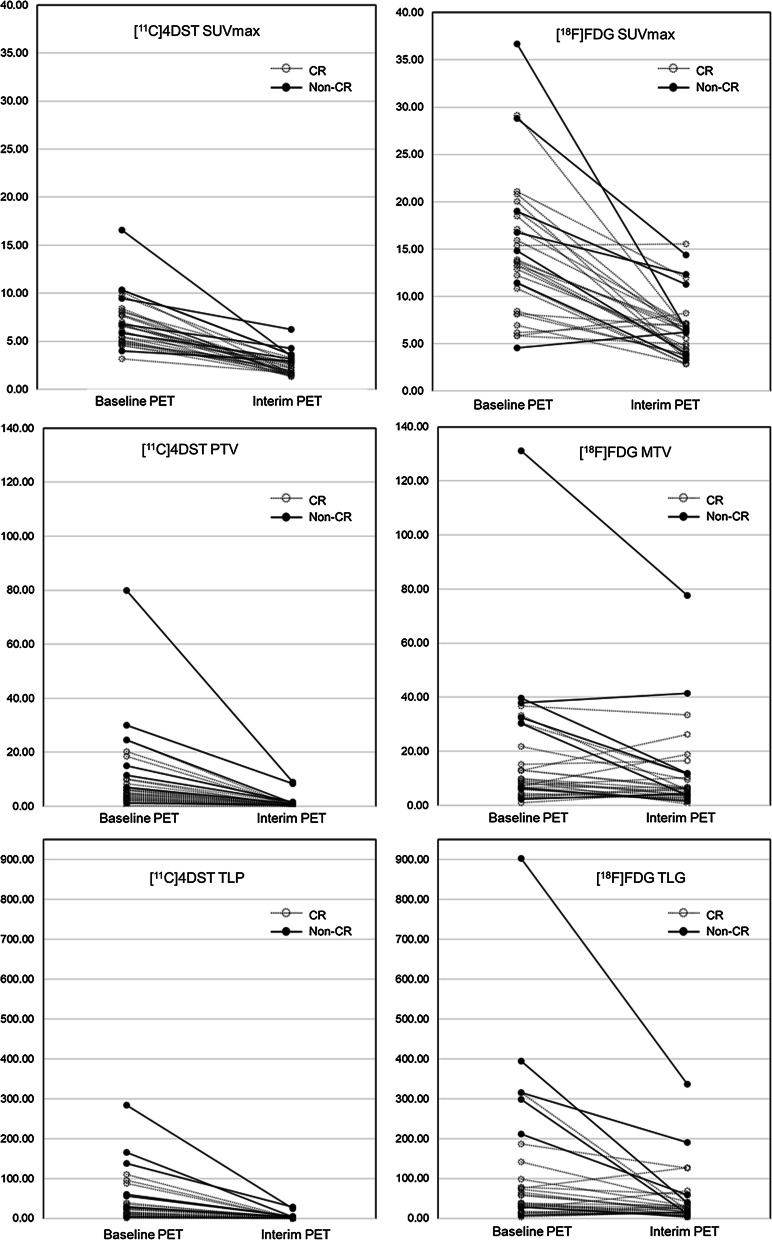
Plot of individual baseline and interim PET parameters, according to the therapy response in 32 patients with HNSCC

Primary tumors were detected in all patients on both the [^11^C]4DST and [^18^F]FDG baseline PET images. All patients showed increased uptake on [^18^F]FDG interim PET images, whereas 18 showed no increased uptake in the primary region on [^11^C]4DST interim PET images. The results in baseline and interim PET parameters are presented in Table 2 and Fig. 1. [^11^C]4DST SUVmax, PTV, and TLP for interim were significantly lower than the corresponding values for baseline (all *P* < 0.001) (Table 2). [^18^F]FDG SUVmax, MTV, and TLG for interim were also significantly lower than the corresponding values for baseline (*P* < 0.001, = 0.02, and = 0.005, respectively).

**Table 2:** Baseline and interim PET parameters for the 32 patients with head and neck squamous cell carcinoma

PET parameter	Baseline PET	Interim PET	*P* value
[^11^C]4DST			
SUVmax	7.01 ± 2.60	2.44 ± 1.05	< 0.001
PTV	10.52 ± 14.79	0.86 ± 2.10	< 0.001
TLP	44.34 ± 59.52	2.58 ± 6.42	< 0.001
[^18^F]FDG			
SUVmax	14.80 ± 7.26	6.50 ± 3.32	< 0.001
MTV	17.49 ± 23.94	11.14 ± 15.27	0.02
TLG	115.39 ± 178.08	44.92 ± 67.38	0.005

Data are given as mean ± standard deviation

SUVmax, maximum standardized uptake value; PTV, proliferative tumor volume; MTV, metabolic tumor volume; TLP, total lesion proliferation; TLG, total lesion glycolysis

### Relation to therapy response

After chemoradiotherapy, 25 patients were found to show CR and 7 non-CR. Table 3 summarizes the results of the association between PET parameters and therapy response. [^11^C]4DST ΔSUVmax, ΔPTV, and ΔTLP for CR group showed significantly greater reduction than the corresponding values for non-CR group (*P* = 0.044, < 0.001, and < 0.001, respectively), whereas there were no significant differences in [^18^F]FDG ΔSUVmax, ΔMTV, or ΔTLG between CR group and non-CR group.

**Table 3:** Association between PET parameters and therapy response for the 32 patients with head and neck squamous cell carcinoma

PET parameter	CR (n = 25)	Non-CR (n = 7)	*P* value
[^11^C]4DST			
ΔSUVmax	− 66.17 ± 12.66	− 50.25 ± 19.63	0.044
ΔPTV	− 97.11 ± 5.23	− 81.77 ± 14.19	< 0.001
ΔTLP	− 97.88 ± 3.84	− 85.63 ± 13.53	< 0.001
[^18^F]FDG			
ΔSUVmax	− 49.23 ± 31.35	− 43.85 ± 40.69	0.90
ΔMTV	4.33 ± 107.57	− 34.69 ± 63.19	0.21
ΔTLG	− 10.71 ± 103.99	− 48.70 ± 70.63	0.18

Data are given as mean ± standard deviation

SUVmax, maximum standardized uptake value; PTV, proliferative tumor volume; MTV, metabolic tumor volume; TLP, total lesion proliferation; TLG, total lesion glycolysis; CR, complete response

**Fig. 2:**
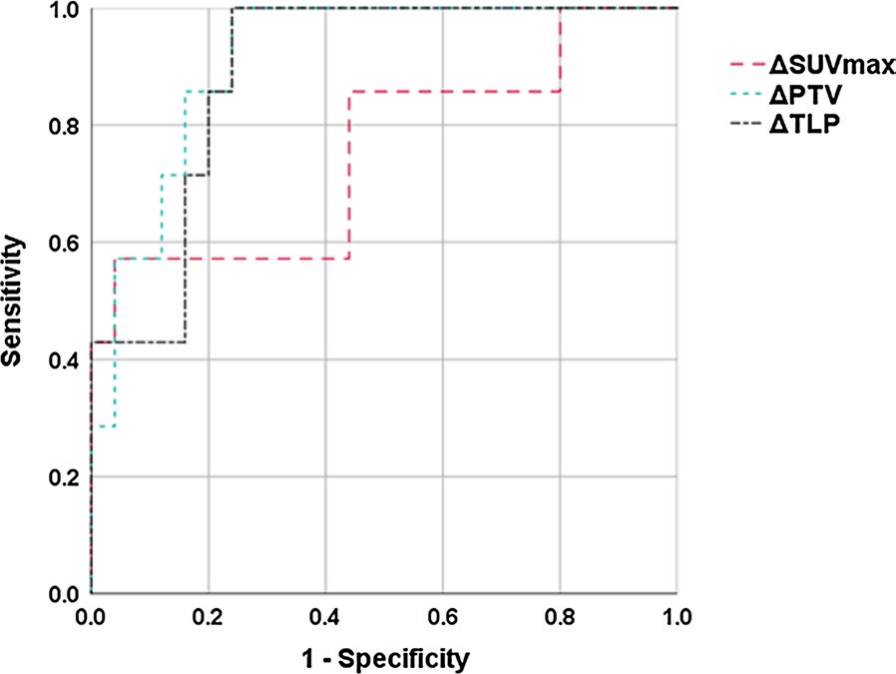
Receiver operating characteristic (ROC) curve analysis for the early identification of patients with non-complete response

[^11^C]4DST ΔSUVmax, ΔPTV, and ΔTLP demonstrated good performance for differentiating the early chemoradiotherapeutic response, with AUC values of 0.75, 0.91, and 0.89, respectively (*P* = 0.044, < 0.001, and = 0.002, respectively) (Table 4 and Fig. 2). [^11^C]4DST ΔPTV of -90 was the best cutoff value for early identification of patients with non-CR. [^18^F]FDG ΔSUVmax, ΔMTV, and ΔTLG demonstrated poor performance for differentiating the early chemoradiotherapeutic response, with AUC values of 0.52, 0.33, and 0.32, respectively (all *P* > 0.05).

**Table 4:** Effectiveness of PET parameters in differentiating the early chemoradiotherapeutic response in patients with head and neck squamous cell carcinoma by receiver operating characteristics (ROC) analysis

PET parameter	AUC (95% CI)	Cutoff value	Sensitivity	Specificity	*P* value
[^11^C]4DST					
ΔSUVmax	0.75 (0.52–0.98)	− 46	0.57	0.96	0.044
ΔPTV	0.91 (0.81–1.00)	− 90	0.85	0.84	< 0.001
ΔTLP	0.89 (0.77–1.00)	− 99	1.00	0.76	0.002
[^18^F]FDG					
ΔSUVmax	0.52 (0.23–0.80)	− 51	0.57	0.72	0.874
ΔMTV	0.33 (0.08–0.59)	7	0.28	0.72	0.194
ΔTLG	0.32 (0.08–0.57)	− 62	0.42	0.48	0.165

SUVmax, maximum standardized uptake value; PTV, proliferative tumor volume; MTV, metabolic tumor volume; TLP, total lesion proliferation; TLG, total lesion glycolysis; AUC, area under the ROC curve; CI, confidence interval

**Fig. 3:**
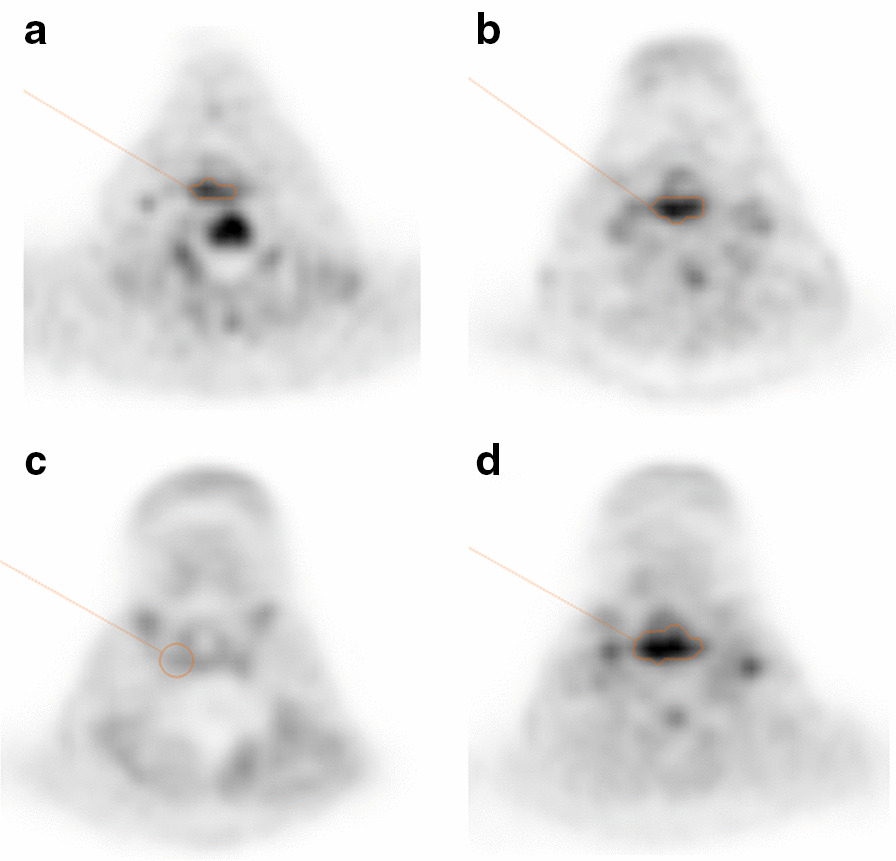
PET images from a 62-year-old male diagnosed with hypopharyngeal squamous cell carcinoma. The orange-contoured area represents the area with an SUVmax greater than 2.5. Pretreatment transverse [^11^C]4DST PET image (**a**) shows an increased uptake in the primary tumor. Pretreatment transverse [^18^F]FDG PET image (**b**) also shows an increased uptake in the primary tumor. Interim (after 46 Gy of radiation) transverse [^11^C]4DST PET image (**c**) shows no abnormal uptake in the primary region. (The orange-contoured area shows the primary region on the basis of baseline PET study.) Interim (after 40 Gy of radiation) transverse [^18^F]FDG PET image (**d**) shows an increased uptake in the hypopharyngeal region. He was classified as showing a complete response at 3 months after treatment

**Fig. 4:**
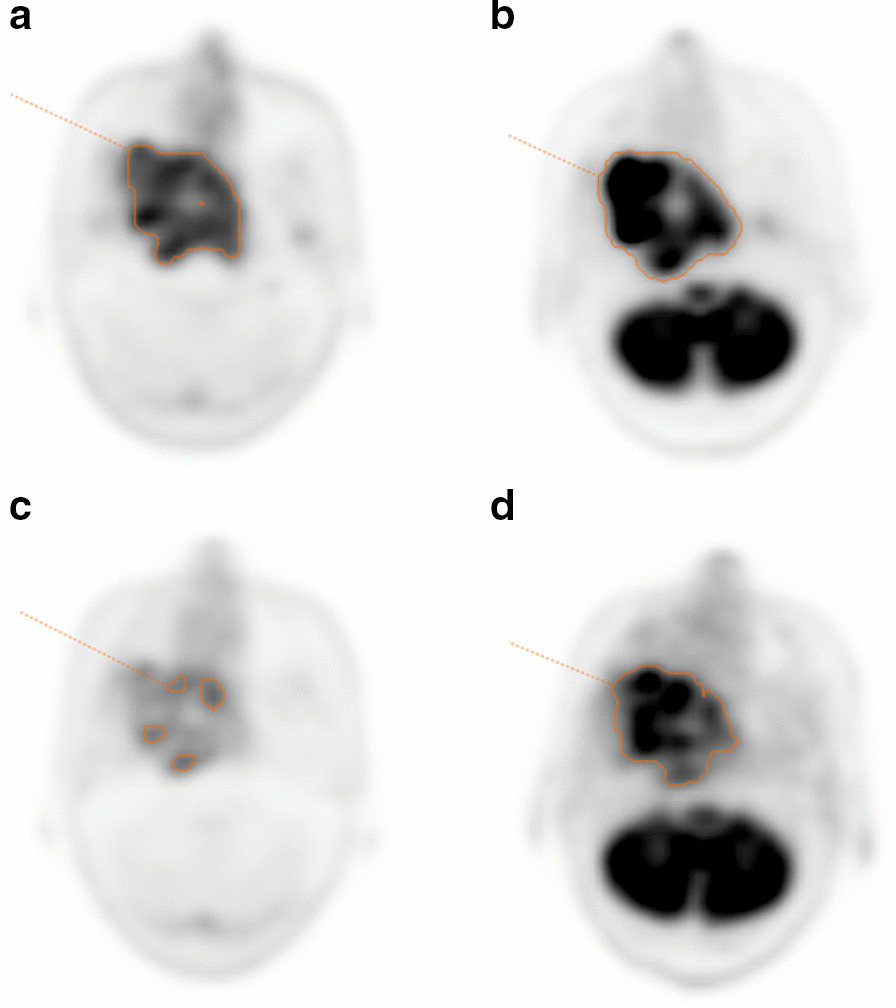
PET images from a 69-year-old female diagnosed with nasopharyngeal squamous cell carcinoma. The orange-contoured area represents the area with an SUVmax greater than 2.5. Pretreatment transverse [^11^C]4DST PET image (**a**) shows an increased uptake in the primary tumor. Pretreatment transverse [^18^F]FDG PET image (**b**) also shows an intense uptake in the primary tumor. Interim (after 44 Gy of radiation) transverse [^11^C]4DST PET image (**c**) shows decreased uptake in the primary region. Interim (after 40 Gy of radiation) transverse [^18^F]FDG PET image (**d**) shows decreased uptake in the primary region. She was classified as showing a non-complete response at 3 months after treatment

Typical PET images from CR and non-CR groups are shown in Figs. 3 and 4, respectively.

## Discussion

It is important to distinguish residual tumors from treatment-induced inflammation to assess the early therapeutic response. Unspecific [^18^F]FDG uptake might persist for the first few posttreatment months, potentially influencing the early evaluation of treatment response [10]. As far as we could determine, this study is the first study focused on [^11^C]4DST PET for early therapeutic assessment in patients with HNSCC, compared to [^18^F]FDG. The present results suggest that interim [^11^C]4DST PET, rather than [^18^F]FDG PET, might be valuable in predicting therapeutic response. Among the three [^11^C]4DST PET parameters, ΔPTV was the best indicator for the early identification of patients with non-CR. Although SUVmax is the most studied PET parameter, some studies have revealed that volumetric information such as MTV and TLG can be a better prognostic indicator of treatment outcomes in head and neck cancers [11].

Ceulemans et al. compared [^18^F]FDG PET during radiotherapy (47 Gy) and 4 months after radiotherapy in patients with HNSCC and concluded that [^18^F]FDG PET during radiotherapy cannot replace post-therapeutic scan due to numerous false-positive results [12]. Kubota et al. reported that a substantial component of [^18^F]FDG uptake in tumor tissue was due to activity localizing in macrophages, young granulation tissue, and other peri-tumoral inflammatory cell elements with greater [^18^F]FDG uptake than tumor cells [13]. In the present study, [^18^F]FDG PET values for ΔSUVmax, ΔMTV, and ΔTLG were not significantly different by treatment response. These results might be a false-positive finding that reflects factors such as treatment-related effects and inflammation other than tumor. However, Hentschel et al. evaluated early interim [^18^F]FDG PET in patients with HNSCC and found that the decrease of SUVmax from before therapy to 1 or 2 weeks (10 or 20 Gy) of chemoradiotherapy was a potential prognostic marker [14]. The appropriate timing of [^18^F]FDG PET during or after chemoradiotherapy remains a topic for further research.

The most direct indicator of proliferation is DNA synthesis that can be measured using radiolabeled thymidine or its analogs. A thymidine analog, 3′-deoxy-3′-[^18^F]fluorothymidine ([^18^F]FLT), has been evaluated in various types of tumor including HNSCC [15, 16]. Kishino et al. compared the feasibility of [^18^F]FLT and [^18^F]FDG PET for evaluation of early locoregional clinical outcomes of chemoradiotherapy in patients with HNSCC and found that the specificity and overall accuracy of [^18^F]FLT PET were significantly higher than those of [^18^F]FDG PET both during radiotherapy (40 Gy) and 5 weeks after [15]. Menda et al. reported a significant reduction in [^18^F]FLT uptake after 10 Gy of radiotherapy in HNSCC [16]. Hoshikawa et al. demonstrated that [^18^F]FLT SUVmax decreased immediately after 30 Gy and no obvious change was found thereafter [17]. In contrast, [^18^F]FDG SUVmax decreased gradually from 30 to 50 Gy, and significant decreases were then observed at the 4- and 6-week time points after radiation [17]. These results suggest that [^18^F]FLT PET may better evaluate the early treatment response than [^18^F]FDG PET.

[^11^C]4DST was developed for cell proliferation imaging and is incorporated into DNA, whereas [^18^F]FLT is not incorporated into DNA [6]. Ito et al. compared pretreatment [^11^C]4DST and [^18^F]FDG PET studies in patients with HNSCC for prediction of recurrence and found that TLP of [^11^C]4DST and TLG of [^18^F]FDG had good prognostic ability for recurrence [8]. Another study demonstrated that [^11^C]4DST PTV and TLP before therapy provided important prognostic information in HNSCC [9]. There are currently no reports on the early assessment of chemoradiotherapy using [^11^C]4DST PET in patients with HNSCC. The present study suggests that [^11^C]4DST PET might assess the early therapeutic response better than [^18^F]FDG PET. The uptake of [^11^C]4DST caused by treatment-induced inflammation is presumed to be lower than that of [^18^F]FDG.

The definition of SUV threshold for volumetric analysis is an important issue. Ito et al. evaluated TLP values with various SUV thresholds that ranged from 2.0 to 5.0 and their results revealed that the ROC curve for TLP as the volume with an SUVmax greater than 2.5 had the highest prognostic ability in patients with HNSCC [8]. Therefore, in the present study, the fixed SUV threshold of 2.5 was chosen. However, the threshold for volumetric analysis has not been established completely.

The current study had some limitations. It was a retrospective design of a small sample size. The results were not internally or externally validated. Primary tumors from various head and neck regions were evaluated and patients had been treated with various therapeutic regimens. Although we have used 40 Gy point for interim PET, the optimal time to perform interim PET remains undecided. We could not assess the lymph nodes other than at the primary site. Quantification was based on PSF-reconstructed data. Rogasch et al. concluded that the use of PSF algorithms for quantitative PET data should be performed with caution—especially if SUV of lesions with high and low contrasts are compared [18]. There is a need to re-evaluate our results with respect to reconstruction parameters. Test–retest reproducibility was not performed here. This is important for treatment response prediction. Rasmussen et al. reported that [^18^F]FDG uptake (SUVmax, MTV, and TLG) in PET/CT was highly reproducible in patients with HNSCC [19]. Few studies have investigated the usefulness of [^11^C]4DST PET in patients with HNSCC. Further additional large prospective studies will be needed to confirm and expand the current results.

Hypoxia induces radioresistance and chemoresistance. In vivo measurement of hypoxia in individual patients is of clinical interest. Okamoto et al. reported that [^18^F]fluoromisonidazole uptake in head and neck cancer rapidly decreased in the early phase of radiotherapy, indicating reoxygenation of the tumor hypoxia [20]. Further studies are needed to assess the usefulness of different PET tracers for early monitoring of the response to therapy.

## Conclusion

The results of this preliminary study suggested that interim [^11^C]4DST PET, rather than [^18^F]FDG PET, might be effective for predicting the chemoradiotherapeutic response in patients with HNSCC.

## Data Availability

All results are provided in the manuscript.
